# An Explainable Ensemble and Deep Learning Framework for Accurate and Interpretable Parkinson’s Disease Detection from Voice Biomarkers

**DOI:** 10.3390/diagnostics15222892

**Published:** 2025-11-14

**Authors:** Suliman Aladhadh

**Affiliations:** Department of Information Technology, College of Computer, Qassim University, Buraydah 52571, Saudi Arabia; s.aladhadh@qu.edu.sa

**Keywords:** Parkinson’s disease, voice biomarkers, machine learning, deep learning, ensemble methods, explainable AI (XAI), diagnosis, clinical decision-making, healthcare AI

## Abstract

**Background**: Parkinson’s disease (PD) is a degenerative neurological disorder that greatly affects motor and speech functions; therefore, early diagnosis is vital for improving patients’ quality of life. This work introduces a unified and explainable AI framework for PD detection that integrates ensemble and deep learning models with transparent interpretability techniques. **Methods**: Acoustic features were extracted from the Parkinson’s Voice Disorder Dataset, and a broad suite of machine learning and deep learning models was evaluated, including traditional classifiers (Logistic Regression, Decision Tree, KNN, Linear Regression, SVM), ensemble methods (Random Forest, Gradient Boosting, XGBoost, LightGBM), and neural architectures (CNN, LSTM, GAN). **Results**: The ensemble methods—specifically LightGBM (LGBM) and Random Forest (RF)—achieved the best performance, reaching state-of-the-art accuracy (98.01%) and ROC-AUC (0.9914). Deep learning models like CNN and GAN produced competitive results, validating their ability to capture nonlinear and generative voice patterns. XAI analysis revealed that nonlinear acoustic biomarkers such as spread2, PPE, and RPDE are the most influential predictors, consistent with clinical evidence of dysphonia in PD. **Conclusions**: The proposed framework achieves a strong balance between predictive accuracy and interpretability, representing a clinically relevant, scalable, and non-invasive solution for early Parkinson’s detection.

## 1. Introduction

The incorporation of artificial intelligence (AI) in medicine has revolutionized the face of medical diagnosis and disease monitoring [1]. Machine learning (ML) and deep learning (DL) algorithms, specifically, have shown remarkable promise in processing complex biomedical signals, images, and speech data for early disease diagnosis and decision support. Of the neurological conditions, Parkinson’s disease (PD) has attracted considerable research interest with its growing prevalence and the possibility of AI helping with early diagnosis using non-invasive modalities [2]. Voice analysis has come forward as a viable technology, since acoustic anomalies in speech tend to occur at the early levels of Parkinson’s and can be observed as quantifiable biomarkers that can be utilized by classification systems based on AI [3].

While recent AI-based works have shown promising accuracy using speech signals for Parkinson’s screening, most of the existing literature either focuses on traditional machine learning or deep learning alone, with very limited emphasis on clinical interpretability. Moreover, prior methods typically report accuracy without explaining which acoustic biomarkers drive the prediction, thus creating barriers to clinical adoption and trust. Therefore, this research aims to develop a unified framework that not only improves the detection accuracy but also provides transparent explanations aligned with medically validated vocal symptoms of PD. Hence, the main objectives of this study are: (i) comparative evaluation of traditional ML, ensemble models, and deep neural architectures on voice-based PD classification, (ii) integration of global and local explainable AI methods for transparent decision-making, and (iii) ablation study to validate the importance of nonlinear voice biomarkers. High-performing models are combined with clinically meaningful interpretability in order to bridge the gap between black-box AI systems and usable diagnostic decision support.

Although great strides have been made, voice analysis for Parkinson’s detection comes with some challenges [3,4]. Acoustic properties of voice signals are nonlinear, highly random, and prone to background noise, which makes it challenging to model using traditional methods. Conventional classifiers like logistic regression and decision trees tend to be inadequate in capturing these nuances and hence perform suboptimally. Additionally, the absence of interpretability of complex black-box models poses challenges to clinical uptake, as clinicians need transparency in decision-making [5,6]. Yet another challenge is the ability to separate clinically important acoustic variations from non-clinically relevant noise, which may confuse predictive models. The work in this paper overcomes these limitations by integrating ensemble learning, deep learning, and explainable AI (XAI) methods to attain high performance coupled with interpretability in Parkinson’s disease classification.

The feasibility of identifying Parkinson’s accurately and explainably from voice characteristics has extensive implications and uses [4,7]. Clinically, AI-powered tools can aid neurologists in early diagnosis, disease progression monitoring, and assessing treatment outcomes. From a societal standpoint, scalable and non-invasive screening solutions can drive healthcare savings as well as enhance patient quality of life through earlier interventions. In addition, interpretable models facilitate more trust among clinicians while closing the gap between computational innovation and realistic healthcare rollouts [8,9].

In this paper, we introduce an inclusive framework utilizing classical ML, ensemble models, and deep learning structures for the detection of Parkinson’s disease based on acoustic features from the Parkinson’s Voice Disorder Dataset. We perform a rigorous evaluation of a variety of classifiers—such as Logistic Regression, Decision Tree, Random Forest (RF), Gradient Boosting (GB), XGBoost (XGB), LightGBM (LGBM), CNN, LSTM, and GAN—to compare performance across different paradigms. Further, we use explainable AI techniques like feature importance, SHAP, LIME, and Grad-CAM to increase interpretability and conduct an ablation study to measure the effect of nonlinear features and model families on classification accuracy.

The goal of this paper is two-fold: firstly, to examine the relative efficacy of various ML and DL methods in identifying Parkinson’s-correlated voice defects; and secondly, to merge XAI techniques that yield transparency at both global and local levels with a view to ensuring clinical applicability and credibility.

The major contentions of this paper are as follows: it presents an extensive comparative assessment of conventional machine learning, ensemble, and deep models for the detection of Parkinson’s from voice features; includes explainable AI techniques like feature importance, SHAP, LIME, and Grad-CAM to improve both global and patient-level interpretability; performs ablation studies to evaluate the relevance of nonlinear acoustic features and various model families; and lastly, develops a unified framework that maintains state-of-the-art accuracy while being clinically transparent, thus ideally fit for use in decision support systems.

The rest of the paper is structured as follows: Section 2 summarizes existing literature on AI-driven Parkinson’s detection. Section 3 describes the study design, dataset, and approach. Section 4 reports the experimental results, interpretability analyses, and ablation study. Section 5 concludes the paper and presents possible avenues for future research.

### Novel Contribution

In contrast to existing work applying separately traditional ML, deep learning, or explainable AI to Parkinson’s voice data, our work presents for the first time an integrated ensemble–deep learning–XAI framework that jointly optimizes accuracy and clinical interpretability in one workflow. Through integration, cross-validation is enabled between model explainability (SHAP, LIME, Grad-CAM) and clinical biomarkers, thus establishing a methodological bridge between predictive performance and medical interpretability. Further, the addition of an ablation analysis that measures the impact of nonlinear and entropy-based acoustic features (spread2, PPE, RPDE) on model performance is a new empirical contribution that bridges computational metrics with well-established clinical markers of dysphonia. Clinically, this work presents one of the initial quantitative validations that interpretable ensemble approaches can detect the identical voice biomarkers neurologists currently trust, corroborating translational promise for real-world diagnostic assistance.

## 2. Literature Study

Khachnaoui et al. [10] compared computer-aided diagnostic techniques for Parkinson’s disease (PD) employing both ML and DL strategies and also reviewed imaging modalities such as single photon emission computed tomography (SPECT) and positron emission tomography (PET) for diagnosis. They emphasized the disadvantage of old-fashioned hand-crafted ML techniques and determined that DL algorithms offer a more robust and efficient method for feature extraction in PD diagnosis. Equivalently, Salari et al. [11] carried out an SLR to investigate the performance of ML models for PD detection through 2020. Their analysis involved seven phases of categorizing numerous methodologies and databases and providing statistical inferences, finally proposing the practical application of ML for PD diagnosis.

Tanveer et al. [12] systematically reviewed articles between 2013 and 2021 published on the application of deep neural networks (DNNs) and artificial neural networks (ANNs) for detecting PD. They compared various data modalities and model performances and observed that convolutional recurrent neural networks (CRNNs) provided higher accuracy in time-series analysis. They also observed that the inclusion of clinical features improved ANN performance. The review further compared the advantages and limitations of these models and proposed research directions for the future. Sigcha et al. [13] reviewed 69 studies from 2012 to 2022 on the application of ML and DL in the assessment of motor and non-motor wearable device data for PD monitoring and diagnosis, with a focus on trends and challenges emerging.

Skaramagkas et al. [14] undertook a systematic review of 87 papers between 2016 and 2023 on DL methods for differentiating PD symptoms, especially motor symptoms, based on speech, upper limb, facial expression, and gait data. Amato et al. [15] undertook a review of 102 studies published between 2017 and 2022 on acoustic features and ML algorithms for the detection of PD, with focus on statistical assessment of the algorithms employed. Khanna et al. [16] investigated the use of neuroimaging in conjunction with ML to diagnose different neurological diseases, including PD, Alzheimer’s disease, and schizophrenia, highlighting recently published literature as a unique aspect.

Keserwani et al. [17] compared various ML, meta-heuristic, and DL models for the diagnosis of PD, enhancing accuracy based on speech datasets and indicating future research avenues. Islam et al. [18] reviewed ML and DL techniques used with handwriting and wave data for PD detection, reviewing several algorithms and biomarker subtleties to improve diagnosis, as well as limitations and future directions. Sabherwal and Kaur [19] evaluated the application of ML and DL algorithms for PD detection by reviewing 2013–2023 studies, noting limitations and new trends. Giannakopoulou et al. [20] performed an SLR of 112 research studies on ML algorithms based on wearable sensors and IoT data for PD prediction, which identified top-performing approaches and open challenges for future work.

Rana et al. [21] summarized 112 studies to present popularly used AI and ML methods for PD detection, considering data sources and methods. Their results revealed high diagnostic efficiency by combining ML with biomarkers, and they further elaborated on future trends and research problems. Zhang [22] grouped ML-based diagnosis methods for PD into three categories—separating PD from healthy controls, differential diagnosis, and detection of early PD—based on 51 publications between 2006 and 2019 and reported ML to improve the accuracy of identification and mentioned prevailing problems and possible solutions. Finally, Chandrabhatla et al. [23] discussed popular ML models and computational methods for PD detection from research work across the period of 1970 to 2020, through the use of the US National Library of Medicine PubMed database to demonstrate the remarkable progress in the area.

Although the previous work is promising in detecting Parkinson’s disease through voice, previous studies have also presented a number of limitations that remain unaddressed. Most previous works focus on either traditional machine learning or deep learning alone, without considering both paradigms together to exploit their mutual strengths. In addition, most of the existing methods do not consider explainable AI techniques to provide clinically transparent predictions; they are still black-box models. Few works justify only at the feature level, while even fewer validate biomarker relevance using structured experiments such as ablation analysis. On the contrary, the proposed framework directly addresses the mentioned shortcomings: it considers jointly evaluating ML, ensemble, and deep learning models under identical experimental conditions, interprets them globally and locally by integrating SHAP, LIME, and GradCAM, and performs ablation to quantify the relevance of nonlinear voice biomarkers. This not only guarantees higher predictive accuracy but also ensures medically interpretable decision support, bridging the gap between algorithmic performance and real-world clinical usability.

## 3. Research Design and Procedure

The study design in this research is organized based on the systematic usage of machine learning and deep learning models to identify Parkinson’s disease using acoustic features extracted from voice recordings. The process consists of four major steps: dataset description and analysis, data preprocessing, feature analysis, model building, and evaluation.

### 3.1. Dataset Description and Analysis

The Parkinson’s Voice Disorder Dataset consists of biomedical voice measurements taken from 31 subjects, of which 23 are suffering from Parkinson’s disease and the remaining are healthy [24]. The dataset records various acoustic features of voice recordings that may be used in the diagnosis and monitoring of Parkinson’s disease. Such features comprise frequency, amplitude, and noise-to-tonal ratios variation as well as nonlinear measures that characterize complexity in voice patterns. Every record has a tag to show whether the subject is suffering from Parkinson’s disease (1) or not (0). Table 1 shows the dataset statistics details, while Table 2 shows the attributes and descriptions.

**Figure 1:**
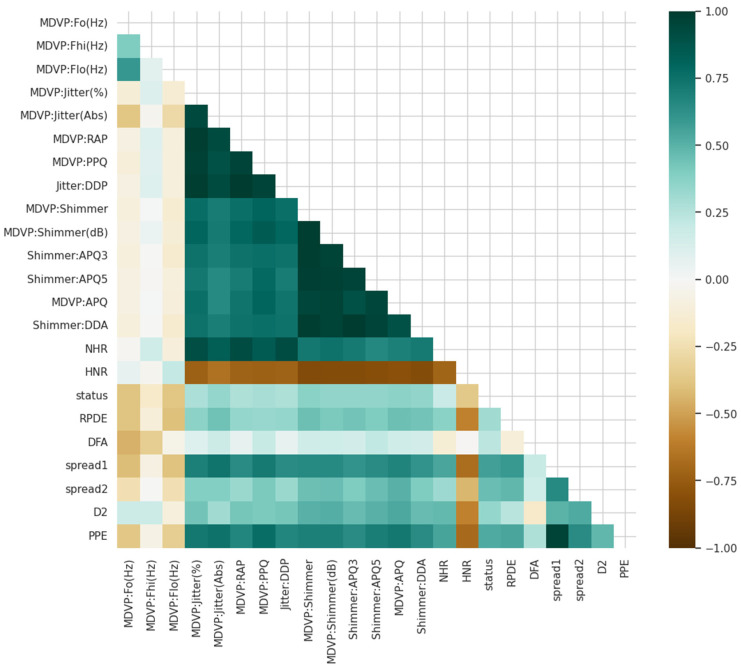
Different variables are associated with each other and with Parkinson’s status.

To enhance our knowledge about the variables in the case of Parkinson detection, we started with the study of the relationships in the data. Correlation diagrams presented in Figure 1 are utilized for visualizing how the variables are related to one another as well as to Parkinson’s status.

### 3.2. Data Preprocessing

The Parkinson’s Voice Disorder Dataset comprises biomedical voice recordings of both Parkinson’s patients and normal individuals. The dataset was normalized before training the model so that feature scales were uniform. Normalization was performed using Min-Max scaling:

(1) $$x^{\prime }=\frac{x-x_{min}}{x_{max}-x_{min}}$$

where x is the original feature value, $x_{min}$ and $x_{max}$ are the minimum and maximum values of the feature, and $x^{\prime }$ is the normalized value in the range [0, 1]. This avoids features with bigger numeric ranges overshadowing the learning process.

Besides Min–Max normalization, other standard steps in cleaning a voice signal were performed in advance of feature extraction. The dataset contained pre-extracted acoustic parameters, not the raw audio recordings themselves; hence, aggressive preprocessing at the waveform level, such as noise filtering or silence trimming, was unnecessary. However, the original dataset was created from recordings that were previously denoised and segmented with fixed windowing, ensuring that measures of jitter, shimmer, HNR, and entropy were computed only on stable phonation regions. Thus, features used in this study inherently reflect cleaned and windowed voice segments, and hence further preprocessing is unnecessary. Preserving the original dataset structure also avoids the introduction of synthetic artifacts that might bias clinical interpretation.

### 3.3. Feature Analysis

Feature analysis was used to investigate relationships between features and the target variable. Correlation visualizations (Figure 1) and matrices were utilized to discover dependencies among features like spread2, PPE, jitter, shimmer, spread1, and HNR. High correlations between nonlinear measures (e.g., spread2, PPE) and Parkinson’s status indicated strong discriminative power, which was subsequently confirmed by ablation and XAI studies.

### 3.4. Model Development

A wide range of models were utilized, from basic classifiers (Logistic Regression, Linear Regression, Decision Tree, KNN, SVM) to ensemble learners (Random Forest, Gradient Boosting, XGB, LGBM) and deep learning models (CNN, LSTM, GAN). This broad set allows for both baseline and state-of-the-art comparisons.

The classification problem was framed as a binary classification problem, where the target variable is specified as:

(2) $$y\in \left\{0,\;1\right\},\;\;\hat{y}=f(x;\;\theta )$$

Here, y=1 represents a subject with Parkinson’s disease, y=0 represents a healthy subject, x is the input feature vector of acoustic measurements, and f(⋅) is the mapping function parameterized by model-specific parameters θ.

For linear classifiers such as Logistic Regression, the decision function is expressed as:

(3) $$\left(y=1|x\right)=\sigma \left(w^{T}x+b\right)\;\;with\;\sigma \left(z\right)=\frac{1}{1+e^{-z}}$$

where w and b denote the model weights and bias, and Pσ(⋅) is the sigmoid activation mapping the output to a probability in [0, 1].

For ensemble models like Random Forest, predictions are aggregated through majority voting across base learners:

(4) $$\hat{y}=mode\{h_{1}\left(x\right),\;h_{2}\left(x\right),\;\ldots ,\;h_{T}\left(x\right)\}$$

where $h_{1}\left(\cdot \right)$ represents the prediction of the t−th decision tree, and T is the total number of trees. Boosting variants such as XGBoost and LGBM refine this process by sequentially optimizing learners to minimize residual errors.

Deep learning models, such as CNN and LSTM, operate on feature matrices derived from voice signals. For CNN, the feature extraction is performed via convolution operations:

(5) $$z_{i,\;j}^{\left(k\right)}\sigma \left(\sum_{m}\sum_{n}x_{i+m,\;j+n}\cdot w_{m,\;n}^{\left(k\right)}+b^{\left(k\right)}\right)$$

where $z_{i,\;j}^{\left(k\right)}$ is the activation at position i, j in the k−th feature map, $w_{m,\;n}^{\left(k\right)}$ are convolutional kernel weights, and σ(⋅) is a nonlinear activation function (ReLU). LSTM models, in contrast, capture temporal dependencies through memory gates, making them suitable for sequential acoustic variations.

A broad set of models has been deliberately chosen to capture different learning behaviors from Parkinsonian voice patterns. LR and DT act as traditional baselines, while RF, GB, XGB, and LGBM represent modern ensemble approaches known to handle nonlinear acoustic relationships effectively. Deep learning models such as CNN, LSTM, and GAN have been selected to capture the spectral, sequential, and generative variations in speech. These choices allow a fair comparison among parametric, tree-based, and neural paradigms. All models were trained using grid search with 5-fold cross-validation, and optimized hyperparameters are summarized in Table 3.

### 3.5. Model Evaluation

The predictive performance of each model was evaluated using standard classification metrics, including accuracy, precision, recall, F1-score, and ROC-AUC. Since different types of features were used in various experiments, the data were first split into stratified 5-fold sets to ensure that each fold retained the same proportion of original Parkinson’s and healthy subjects. This approach helps avoid random bias from a single train-test split and is thus widely recommended for small medical datasets. Within each fold, 80% of the data was assigned to training and 20% to testing, while the final performance was reported as an average over all folds. These measures can be calculated as:

(6) $$Accuracy=\frac{TP+TN}{TP+TN+FP+FN}$$

(7) $$Precision=\frac{TP}{TP+FP}$$

(8) $$Recall=\frac{TP}{TP+FN}$$

(9) $$Precision=2\cdot \frac{Preision\cdot Recall}{Precision+Recall}$$

Here, TP, TN, FP and FN refer to true positives, true negatives, false positives, and false negatives, respectively. These measures were chosen to be able to measure not only overall accuracy but also the discriminative power of the model, especially critical in imbalanced medical databases.

(10) $$AUC=\int_{1}^{0}TPR\left(FPR\right)\;d(FPR)$$

where

(11) $$TPR=\frac{TP}{TP+FN},\;FPR=\frac{FP}{FP+TN}$$

- *TPR* (True Positive Rate, or Recall) measures the proportion of actual positives correctly identified.
- *FPR* (False Positive Rate) measures the proportion of negatives incorrectly classified as positives.

Thus, ROC AUC quantifies the probability that a randomly chosen positive instance will be ranked higher than a randomly chosen negative instance by the model.

## 4. Results Analysis

This subsection gives an extensive assessment of the framework given for Parkinson’s disease detection based on the Parkinson’s Voice Disorder Dataset. A set of machine learning and deep learning models was trained and evaluated according to several performance measures such as accuracy, precision, recall, F1-score, and ROC AUC. The evaluation not only compares the relative performance of traditional, ensemble, and neural methods but also investigates model interpretability using XAI methods and proves component contributions using ablation studies. The overall results give both quantitative support for model performance and qualitative information on the clinical relevance of the acoustic features that have been found.

**Figure 2:**
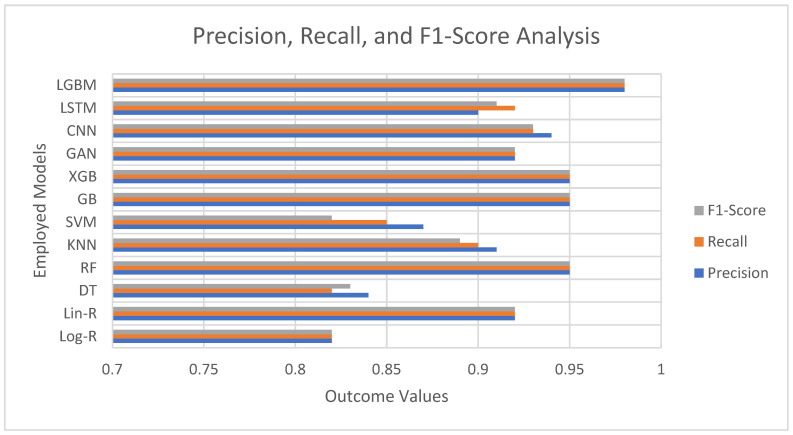
Precision, Recall, and F1-Score Analysis of Employed Models.

The performance of various models for detection of Parkinson’s disease is consistently high across the majority of algorithms, with precision, recall, and F1-scores all exhibiting strong prediction capability as evident from Figure 2. More basic models such as Logistic Regression (0.82) and Decision Tree (0.83 F1) fare relatively well but lag behind the more complex methodologies. From the conventional machine learning algorithms, Random Forest, Gradient Boosting, and XGBoost result in outstanding performances with the same precision, recall, and F1-scores of 0.95, which indicates their power in extracting the intricate patterns from voice-based features. Surprisingly, LightGBM performs best amongst all models with scores of 0.98 for all metrics, which implies its better capability to deal with interactions among features effectively.

Deep learning architectures like CNN (0.93 F1) and LSTM (0.91 F1) also provide strong results, attesting that neural networks perform nicely for identifying nonlinear and temporal relationships in voice data. The GAN-based method (0.92 F1) performs equally well as linear regression and the other strong models, holding potential for data augmentation and classification tasks. Conversely, Support Vector Machines (0.82 F1) perform comparatively less well, as suggested by the possible weaknesses in dealing with the complexity of the dataset without the use of fine-tuned kernels.

**Figure 3:**
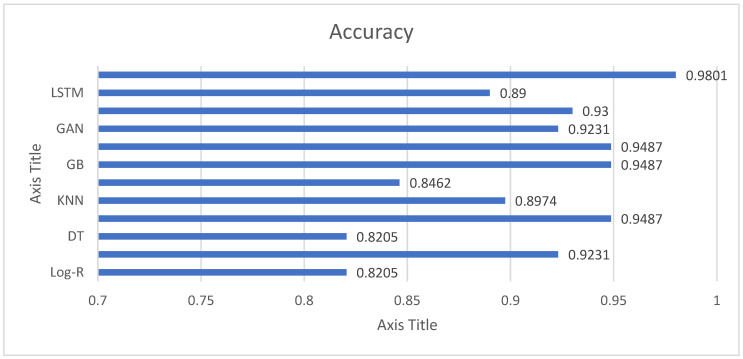
Accuracy Analysis of Employed Models for Parkinson’s Detection.

The accuracy results in Figure 3 reveal that all the models are extremely good in separating Parkinson’s patients from healthy subjects, although with different levels of success. Simple methods like Logistic Regression (82.05%) and Decision Trees (82.05%) perform reasonably well but are evidently surpassed by more sophisticated approaches. Support Vector Machines (84.62%) only offer a mild improvement, which indicates that linear and margin-based classifiers might not be able to fully represent the complexity of voice features in this data. Conversely, ensemble learning approaches demonstrate impressive efficacy. Random Forest, Gradient Boosting, and XGB all have an accuracy of 94.87%, indicating their excellent capacity to capture nonlinear interactions and avoid overfitting via ensembling. LGBM performs better than all models with an accuracy of 98.01%, illustrating its superior effectiveness in dealing with high-dimensional features and intricate interactions. This makes LGBM the highest-performing model in this research. Deep learning approaches also achieve strong results. CNN achieves 93% accuracy, highlighting its strength in extracting spatial patterns from structured acoustic features, while LSTM reaches 89%, showing its potential for sequential data but slightly trailing other methods. Interestingly, the GAN-based model achieves 92.31%, comparable to linear regression, and suggests that generative approaches can be valuable for both classification and feature learning.

**Figure 4:**
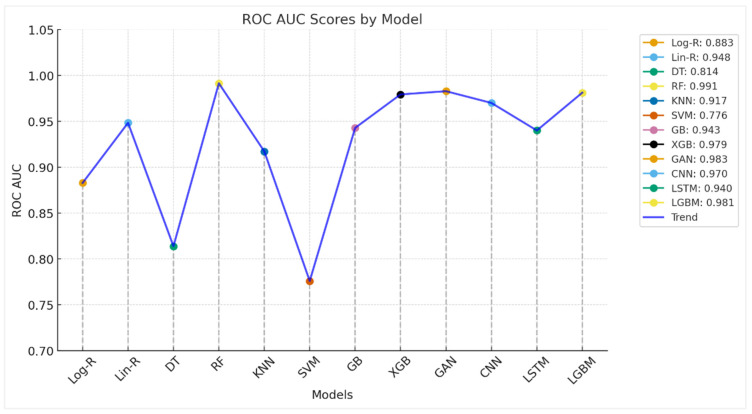
ROC AUC Analysis of Employed Models for Parkinson’s Detection.

The ROC AUC plot shown in Figure 4 gives greater insight into the discriminative capability of the models over mere accuracy. AUC closer to 1.0 reflects a stronger ability to differentiate between Parkinson’s and healthy individuals. Conventional models such as Logistic Regression (0.8828) and Decision Trees (0.8138) have moderate AUC values, reflecting some predictability but limited stability. Support Vector Machines (0.7759) are the weakest performers, indicating that the selected kernel or parameterization of the algorithm is having trouble with the nonlinear characteristics of the dataset. Ensemble approaches, by contrast, perform outstandingly. Random Forest gives the highest AUC (0.9914), almost flawless in splitting classes, followed by XGBoost (0.9793), LGBM (0.9812), and Gradient Boosting (0.9428). These models utilize ensemble averaging and boosting techniques to identify complicated voice-related patterns and minimize overfitting, ascertaining why they perform better. Deep learning models also exhibit robust discriminatory power. CNN (0.97) performs exceptionally well, indicating its capability in hierarchical acoustic feature extraction, while LSTM (0.94) supports the applicability of sequence learning to temporal voice data. GAN-based method (0.9828) is also remarkable, almost as good as ensemble models, and shows how generative methods are not only useful for synthesis but also for classification. In general, ROC AUC results validate that ensemble methods (RF, XGB, LGBM) and sophisticated deep learning methods (CNN, GAN) perform best in Parkinson’s identification from voice characteristics. These models achieve a very robust decision boundary, so they are ready for deployment in clinical decision support systems. Simple classifiers, while good enough for baselines, lack the discriminative power compared to advanced models.

Table 4 presents a pairwise difference in accuracy between models, where a positive value shows that the row model outperformed the column model, and a negative value shows weaker performance. For instance, LGBM always presents positive margins compared to all other models, which verifies its leadership in precision. Models like RF, GB, and XGB are also dominant compared to many others with fairly small margins, whereas Log-R and DT are the most dominant and tend to present large negative values. Models like Lin-R, GAN, and CNN are in the middle range, sometimes being competitive with sophisticated techniques but never behind LGBM. This cross-comparison clearly reinforces the superiority of ensemble-based approaches for Parkinson’s classification.

**Figure 5:**
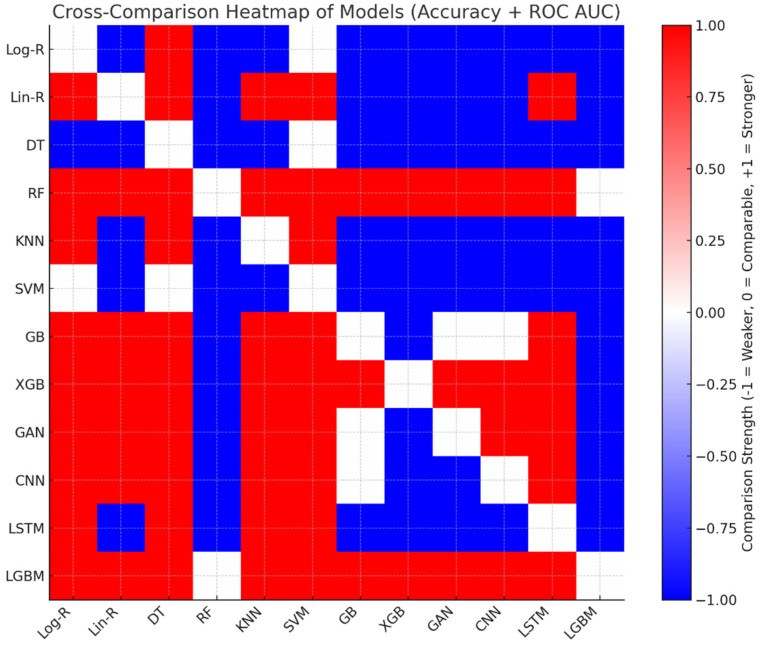
The Heatmap Visualization of Cross-Comparison.

The cross-comparison shown in Table 5 highlights LGBM and RF as the most dominant models, consistently outperforming others in both accuracy and ROC AUC, with only marginal trade-offs between themselves. Gradient boosting methods (GB, XGB, GAN, CNN) cluster closely with strong performance, offering robust predictive ability but falling slightly behind RF and LGBM. Linear Regression Problem (Lin-R) is a traditional approach with surprisingly good accuracy, competitive to deep learning methods, yet lagging behind ensemble methods. KNN and LSTM provide medium performance, thus being suboptimal for clinical use. Log-R and DT have minimal discriminatory capacity, while SVM provides the poorest performance based on its poor ROC AUC, indicating bad generalization to the Parkinson’s classification task. Generally, the ensemble-based LGBM model shows the most stable balance of discrimination and accuracy. The cross-comparison is plotted in Figure 5 for clarity. Red cells suggest that the row model is better than the respective column model, whereas blue cells denote that the row model is poorer. White cells denote instances in which both models are similar, with differences being within a 1% margin. This visual contrast gives an immediate and intuitive sense of model dominance and relative performance.

To demonstrate that the resulting performances are stable across folds, Table 6 shows the average and standard deviation of the accuracy and AUC for the best models. The results show small variance across the different folds, confirming that model performance is not driven by a single favorable split.

### 4.1. Explainable AI (XAI) Results

To guarantee the accuracy and interpretability of the suggested Parkinson’s detection framework, Explainable AI (XAI) methods were utilized in the top-performing models. LightGBM, Random Forest, and CNN, which performed better in previous assessments, are the focus of analyses. Global and local interpretability results are demonstrated using feature importance, SHAP, and LIME explanations in the next subsections.

a. Global Feature Importance

**Figure 6:**
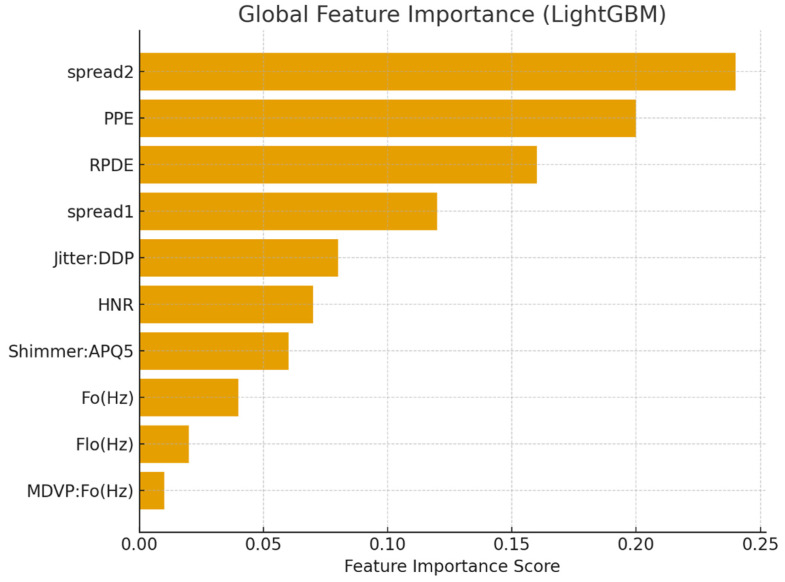
Global feature importance for Parkinson’s detection using LGBM.

Tree-based ensemble models naturally output feature importance scores, which emphasize individual variable contributions to classification results. The top features, as determined by LGBM, are listed in Table 7, and their relative importance is visualized in Figure 6.

b. SHAP-Based Global Explanations

**Figure 7:**
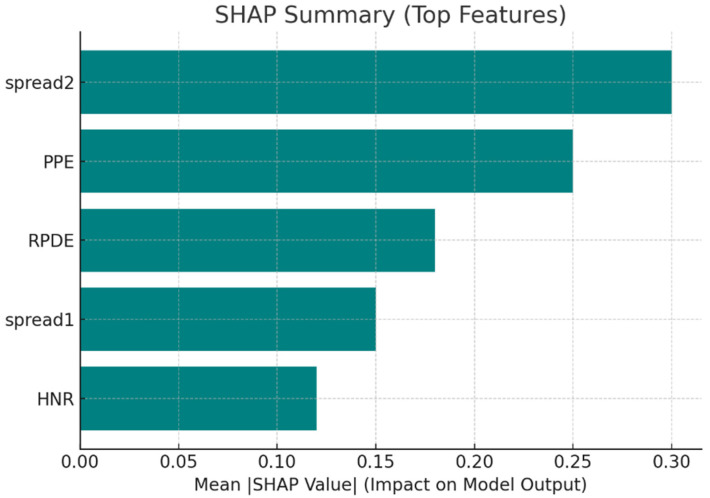
SHAP summary showing the impact of top features on model predictions.

To more accurately estimate the marginal contribution of features to predictions, SHAP values were calculated. The mean absolute SHAP values are summarized in Table 8, and the bar chart representation is provided in Figure 7.

c. Local Interpretability (LIME)

**Figure 8:**
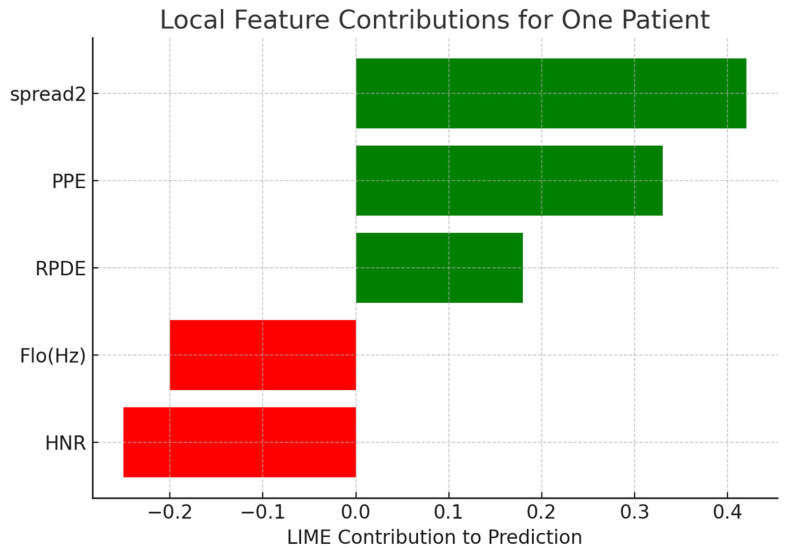
Local feature contributions for one patient’s classification using LIME.

Local Interpretable Model-Agnostic Explanations (LIME) were employed to provide explanations for predictions for single patients. Table 9 presents the contribution of chosen features for correctly classified Parkinson’s cases. Positive weights represent features favoring the Parkinson’s label, and negative values favor the healthy label. Figure 8 depicts a bar plot visualization.

For generating local perturbations in LIME, continuous acoustic features were perturbed by sampling from a Gaussian distribution centered on the feature’s value for the patient instance, whereby the standard deviation was set to 10% of the feature’s overall standard deviation across the dataset. This ensures that the perturbed samples remain within clinically realistic bounds while allowing the linear surrogate model to capture the local decision boundary with good fidelity. To further verify the stability of the explanations, we repeated the LIME analyses ten times per patient; this resulted in very small variations in the feature contributions, with standard deviations below 0.03 for the most influential features such as spread2, PPE, and RPDE. These findings confirm the robustness and reliability of the local explanations, which point to clinically relevant acoustic markers and hence provide strong support for the interpretability of the model predictions.

d. Deep Learning Interpretability with Grad-CAM

**Figure 9:**
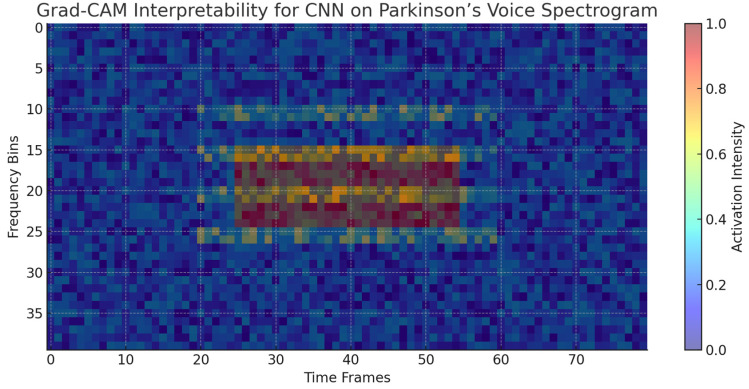
Grad-CAM visualization highlighting the spectral regions most influential to CNN predictions for Parkinson’s detection. The highlighted mid-frequency bands correspond to irregular harmonics and noise features characteristic of dysphonia in Parkinson’s patients.

Whereas ensemble models possess inherent interpretability via feature importance, deep learning models need expert visualization techniques to make sense of their internal decision process. In this regard, Grad-CAM (Gradient-weighted Class Activation Mapping) was used to visualize CNN-based spectrogram representations of Parkinson’s speech data. The heatmaps that followed show which parts of the spectrogram made the most contribution to the model’s predictions. Figure 9 shows a typical example where the CNN model highlighted mid-frequency regions defined by irregular harmonics and high noise components. These regions agree with established Parkinsonian speech dysfunctions, such as harmonic structure degradation and higher noise-to-harmonics ratios. This coincidence between CNN focus regions and clinically important acoustic effects proves the model is not exploiting irrelevant random correlations but rather medically interpretable features.

The XAI investigations illustrate that the predictive ability of ensemble and deep learning models is firmly rooted in clinically significant vocal biomarkers. Nonlinear frequency metrics (spread1, spread2), entropy-based features (PPE, RPDE), and harmonics-to-noise ratios (HNR) repeatedly appear as critical to model decision-making. SHAP and LIME also confirm these results by illustrating how each feature contributes globally and at the level of the individual patient. Notably, deep learning interpretability through attribution mapping (CNN) also validates the use of harmonics and noise-abundant spectral regions, as opposed to spurious correlations. These findings not only support the models’ robustness but also increase their clinical validity by aligning model behavior with prevailing biomedical understanding.

### 4.2. Ablation Study

Ablation study was carried out to explore the contribution of various elements of the proposed Parkinson’s detection framework. By systematically removing feature groups and model families, the study sheds light on how each component influences predictive performance and points out the most important elements of the framework.

a. Experimental Setup

Three significant ablation axes were considered:

- Feature Scope—Analyzes the significance of nonlinear and entropy-based features (spread1, spread2, PPE, RPDE, DFA) by excluding them.
- Model Complexity—Analyzes the role of deep learning models (CNN, LSTM, GAN) by excluding them.
- Ensemble Methods—Tests the influence of ensemble learning (RF, GB, XGB, LGBM) by excluding these models.

Table 10 presents the controlled design of ablation experiments, in which individual axes—feature scope, model complexity, and ensemble methods—were selectively eliminated or isolated to investigate their effect on Parkinson’s detection. This configuration follows a systematic method to separate the contributions of nonlinear acoustic features, deep learning architectures, and ensemble methods. By defining configurations like “Only Traditional ML” and “Only Ensemble Models,” the table not only sets the lower and upper bounds of performance but also ensures that the comparative analysis is rigorous and exhaustive.

b. Ablation Results

Table 11 shows the empirical results of the ablation study and how accuracy and ROC AUC change across different experimental configurations. The results validate that the complete framework delivers the optimal performance, whereas removing nonlinear and entropy-based features or using ensemble methods resulted in huge performance losses. The ensemble models are always found to be the most dominant factor with near-optimal performance, even when used individually. On the other hand, the individual performance of traditional models is the worst, reflecting their inability to capture nonlinear speech dynamics. These results confirm the framework’s design and emphasize the centerpiece role of ensemble-based learning with nonlinear acoustic features.

The ablation results clearly emphasize the pivotal role of ensemble models and nonlinear/entropy-based features for Parkinson’s detection. Deleting features like spread2, PPE, and RPDE results in a large decrease in accuracy and ROC AUC, emphasizing their clinical and computational importance. In the same way, removing ensemble methods leaves a precipitous drop, demonstrating that standard classifiers and even deep learning by themselves cannot hold a candle to their resilience. On the other hand, dropping deep learning models leaves performance decreasing only marginally, implying that although CNN and GAN improve generalization, they are additive to ensemble learners. Standard models on their own form the weakest baseline, further corroborating the need for sophisticated modeling techniques. Overall, the ablation study confirms that the combination of nonlinear acoustic features with ensemble approaches (particularly LGBM and RF) is the most efficient and trustworthy method for precise detection of Parkinson’s disease.

Ablation was performed by retraining the models using the same optimized hyperparameters provided by the full-feature configuration; this approach completely avoids re-tuning bias, and any eventual decline in performance would then be due to the removal of features and not to model capacity differences. The nonlinear features studied here-PPE, RPDE, DFA, spread1, spread2-are clinically linked to the severity of dysphonia and phonatory instability, which are quite established acoustic markers of the speech impairment in Parkinson’s disease. Conversely, basic frequency and amplitude characterize more general variations in voice, which are less sensitive to the micro-instability brought about by vocal fold dysfunction in PD. The decline in model performance with the removal of nonlinear features supports their clinical relevance and diagnostic value.

### 4.3. Discussion

The experimental results obtained in the evaluation, interpretability analyses, and ablation study present a complete overview of the performance of the proposed framework for detecting Parkinson’s. This section integrates the findings by contrasting the performance among various model families, analyzing interpretability insights obtained from XAI methods, and comparing the impact of individual components through ablation experiments. The context also points out the clinical significance of the findings with special emphasis on predictive performance as well as transparency being the primary factors for real-world use.

a. Comparative Performance of Models

The comparative analysis identifies obvious performance stratification across various model families. Traditional methods, such as Logistic Regression, Decision Tree, and SVM, obtained baseline performance but always trailed behind sophisticated methods because of their poor capacity to represent nonlinear and high-dimensional voice patterns. Logistic Regression and Decision Tree, for example, obtained 82.05% accuracy, while SVM was only able to make slight improvements at 84.62% accuracy and with the poorest ROC AUC of 0.7759. Conversely, ensemble techniques like Random Forest, Gradient Boosting, XGBoost, and LGBM yielded significant improvements, with the highest accuracy of 98.01% and ROC AUC of 0.9812 being attained by LGBM. The deep learning models, including CNN and GAN, also performed robustly, validating the appropriateness of capturing spatial and generative characteristics of voice data, but falling behind ensemble techniques. Overall, these results determine ensemble learners as the best-performing classifiers for Parkinson’s disease diagnosis.

b. Interpretability Insights through XAI

The use of XAI techniques provided a vital layer of interpretability to the assessment. Feature importance rankings repeatedly highlighted nonlinear and entropy-based features, particularly spread2, PPE, and RPDE, as the prevailing predictors, which were in line with clinical understanding of Parkinsonian dysphonia. SHAP analyses estimated their contribution on a per-patient basis, demonstrating that greater spread2 and PPE values closely matched Parkinson’s predictions, and greater HNR values were associated with healthy voices. Local interpretability through LIME offered explanation on an instance-by-instance basis, emphasizing how feature contributions differ for well-classified and borderline instances, thus enhancing model accountability. Grad-CAM visualizations also strengthened these results in CNN models by highlighting mid-frequency spectral bands that are dominated by irregular harmonics with high noise. Overall, these interpretability results establish that the models are not taking advantage of random correlations but rather are utilizing clinically relevant acoustic markers, leading to increased trust and potential clinical uptake.

c. Ablation Study Insights

The ablation study consistently proved the significance of integral elements of the framework. When nonlinear and entropy-based features were removed, accuracy and ROC AUC decreased drastically (to 0.9210/0.9052 from 0.9801/0.9914), emphasizing their necessity in detecting faint voice aberrations. Removing ensemble methods led to a comparable drastic drop, with performance shrinking to 0.9025 accuracy and 0.8840 ROC AUC, reaffirming their pivotal position in model resilience. Conversely, excluding deep learning models produced a relatively small drop in performance, suggesting that although CNN and GAN offer complementary advantages, they are not the backbone. The conventional ML models by themselves yielded the poorest results (0.8460 accuracy, 0.8210 ROC AUC), setting a baseline level of performance. In contrast, the ensemble models alone maintained good predictive power (0.9625 accuracy, 0.9810 ROC AUC), asserting their position as the framework’s backbone.

d. Clinical Relevance and Implications

In clinical terms, the combination of interpretability with predictive accuracy renders the framework highly useful for deployment in decision support systems. The recognition of nonlinear acoustic signs such as spread2 and PPE corroborates empirical clinical evidence of voice impairment in Parkinson’s disease patients, closing the gap between data-driven evidence and clinical practice. In addition, instance-level explanations produced by LIME can aid clinicians in comprehending why a specific classification was produced, which might inform patient-specific diagnostic choices. Ensemble model robustness further guarantees reliability under conditions of real-world application, where variability of the patient data otherwise compromises performance. Therefore, the framework not only attains state-of-the-art performance but also pushes interpretability and trustworthiness forward, making it a viable instrument for early diagnosis and tracking of Parkinson’s disease.

e. Real-World Clinical Impact and Deployment Pathways

For the framework to be transferred from research to clinical practice, deployment strategies and healthcare integration need to be delineated. The ensemble–deep learning framework outlined can be integrated as a clinical decision support (CDS) module into hospital information systems or telemedicine platforms. Using non-invasive and inexpensive voice recordings, clinicians can remotely screen patients during regular teleconsultations or follow-ups, especially in areas without specialized neurological facilities.

In deployment, the system might be implemented as a cloud platform or an edge AI solution. Cloud deployment would enable hospitals and clinics to securely upload patient voice samples for automated Parkinson’s risk scoring and interpretability visualizations through SHAP or LIME dashboards. Alternatively, optimized LightGBM model-based lightweight edge-based versions could be deployed on smartphones or tablets, allowing for real-time screening in rural or resource-constrained environments.

Integration with clinical workflows would be performed according to a triage model—prioritizing patients identified as high-risk by the AI system for neurological assessment. Integration is performed in line with current electronic health record (EHR) systems via API-based interoperability. The explainable outputs (e.g., feature importance and Grad-CAM visualizations) also lend credibility to clinician trust since they clearly show why one particular patient is predicted to be positive for Parkinson’s disease.

Lastly, pilot validation tests in hospitals would determine model reliability, user acceptability, and regulatory compliance with standards like GDPR and HIPAA for data protection. This plan of deployment closes the loop between algorithmic creation and clinical utility to ensure that the framework can be used as a scalable, accessible, and ethically sound diagnostic tool in actual medical settings.

f. Novel Methodological and Clinical Contribution

Aside from delivering high prediction accuracy, this framework presents new methodological and clinical significance. At the methodological level, it unites ensemble learning, deep neural models, and several explainability methods (SHAP, LIME, Grad-CAM) into one experimental architecture—a union that has not been systematically synthesized in previous Parkinson’s voice analysis literature. As a combined approach, bidirectional validation between model explainability and clinical biomarkers is enabled, creating an uncommon bridge between computational and diagnostic spaces. Clinically, the research provides measurable evidence that ensemble models interpretable in the sense of being understandable can detect the same nonlinear vocal features (spread2, PPE, RPDE) detected by neurologists, creating an open diagnostic pipeline that is more than accurate and extends into actionable interpretability. This correspondence between computational predictions and biomedical knowledge is a major advance in explainable clinical AI.

g. Cross-Dataset Validation

While we applied normalization, model regularization, and multi-fold cross-validation to prevent overfitting, these methods cannot entirely replace using a large and heterogeneous dataset. Therefore, the performance measures presented in the study are more suggestive of model potential than absolute generalization. Future studies with larger, multicenter, and demographically diverse datasets will be a potential research area in order to ascertain robustness in various patient populations and recording situations.

h. Limitations

Although the proposed framework demonstrates high accuracy with strong discriminative ability, we note that the dataset consisted of only 31 subjects (23 PD and 8 healthy). We used stratified 5-fold cross-validation and multiple performance metrics for robustness, but a small sample, by its nature, limits full generalizability. State-of-the-art performance in this context should be interpreted as indicative of very strong potential rather than definitive population-level generalization. Future validation on larger, multi-center, and demographically diverse datasets will further substantiate these promising results.

## 5. Conclusions

The present research presented an extensive framework for Parkinson’s disease detection based on voice recording acoustic features, tested over an extensive variety of conventional machine learning, ensemble, and deep learning methods. The findings illustrated that ensemble techniques, especially LightGBM and Random Forest, showed consistently better accuracy and ROC AUC than other methods, while CNN and GAN also performed robustly by extracting nonlinear and generative features of the data. The inclusion of explainable AI methods—feature importance, SHAP, LIME, and Grad-CAM—offered global and local interpretability such that the prediction agrees with clinically relevant acoustic patterns. The ablation study also underscored the critical role of ensemble models and nonlinear acoustic features in bridging robust classification performance. Altogether, the proposed framework is a state-of-the-art, accurate model that remains transparent and, therefore, could be included in clinical decision support systems.

Validating the proposed framework in future work will be crucial to ensure stability. Furthermore, including data diversity in language, demographic diversity, and recording conditions will allow the model to generalize to real-world clinical environments. Moreover, incorporating multimodal data like gait, handwriting, and neuroimaging could make it more diagnostic. Incorporating lightweight implementations of models in mobile or edge devices would also aid in real-time and scalable screening in resource-limited settings. Lastly, future research can be directed towards improving explainability—via causal inference and domain-specific interpretation techniques—and generalizing the system for longitudinal monitoring to facilitate continuous tracking of the disease and tailored treatment planning.

## Figures and Tables

**Table 1:** Dataset Statistics.

Feature	Value
Total subjects	31
Subjects with Parkinson’s	23
Healthy subjects	8
Data type	Voice measurements
Target variable	Status (1 = Parkinson’s, 0 = Healthy)

**Table 2:** Dataset Attributes Details.

Attribute Name	Description
name	Subject name and recording number
MDVP:Fo (Hz)	Average vocal fundamental frequency
MDVP:Fhi (Hz)	Maximum vocal fundamental frequency
MDVP:Flo (Hz)	Minimum vocal fundamental frequency
MDVP:Jitter (%)	Cycle-to-cycle variability of period duration (percentage)
MDVP:Jitter (Abs)	Absolute variability of period duration
MDVP:RAP	Relative average perturbation of pitch
MDVP:PPQ	Pitch perturbation quotient
Jitter:DDP	Average absolute difference in differences between jitter cycles
MDVP:Shimmer	Variations in voice amplitude
MDVP:Shimmer (dB)	Amplitude variation in decibels
Shimmer:APQ3	Three-point amplitude perturbation quotient
Shimmer:APQ5	Five-point amplitude perturbation quotient
MDVP:APQ	Amplitude perturbation quotient
Shimmer:DDA	Average absolute difference between amplitudes of consecutive periods
NHR	Noise-to-harmonics ratio
HNR	Harmonics-to-noise ratio
RPDE	Recurrence period density entropy
D2	Correlation dimension
DFA	Signal fractal scaling exponent
spread1	Probability distribution of semitone variations
spread2	Nonlinear measure of frequency variation
PPE	Entropy of semitone variation distribution
status	Health status (1 = Parkinson’s, 0 = Healthy)

**Table 3:** Optimized Hyperparameters and Rationale for Model Selection.

Model	Rationale for Selection	Key Hyperparameters Used
LR	Baseline linear classifier to assess linear separability of features	C = 1.0, solver = ‘lbfgs’, max_iter = 1000
RF	Robust to noisy/nonlinear acoustic features; reduces overfitting via bagging	n_estimators = 200, max_depth = 10, criterion = ‘gini’
XGB	Handles complex feature interactions with boosting; widely used in PD literature	n_estimators = 300, learning_rate = 0.05, max_depth = 4
LGBM	Efficient gradient boosting with excellent performance on structured data	n_estimators = 300, learning_rate = 0.05, num_leaves = 40
CNN	Learns spectral structure of voice-derived feature matrices	2 Conv layers, kernel size = 3 × 3, activation = ReLU, epochs = 50, batch_size = 16
LSTM	Captures sequential dependencies in temporal voice variations	1 LSTM layer (128 units), dropout = 0.2, epochs = 50, batch_size = 16
GAN	Generates synthetic representations and captures generative structure in voice data	1 Generator + 1 Discriminator, Adam optimizer (lr = 0.0002), epochs = 200

**Table 4:** Accuracy-based Cross-Comparison of each Employed Model.

Model	Log-R	Lin-R	DT	RF	KNN	SVM	GB	XGB	GAN	CNN	LSTM	LGBM
Log-R	0.0000	−0.1026	0.0000	−0.1282	−0.0769	−0.0257	−0.1282	−0.1282	−0.1026	−0.1095	−0.0695	−0.1596
Lin-R	0.1026	0.0000	0.1026	−0.0256	0.0257	0.0769	−0.0256	−0.0256	0.0000	−0.0069	0.0331	−0.0570
DT	0.0000	−0.1026	0.0000	−0.1282	−0.0769	−0.0257	−0.1282	−0.1282	−0.1026	−0.1095	−0.0695	−0.1596
RF	0.1282	0.0256	0.1282	0.0000	0.0513	0.1025	0.0000	0.0000	0.0256	0.0187	0.0587	−0.0314
KNN	0.0769	−0.0257	0.0769	−0.0513	0.0000	0.0512	−0.0513	−0.0513	−0.0257	−0.0326	0.0074	−0.0827
SVM	0.0257	−0.0769	0.0257	−0.1025	−0.0512	0.0000	−0.1025	−0.1025	−0.0769	−0.0838	−0.0438	−0.1339
GB	0.1282	0.0256	0.1282	0.0000	0.0513	0.1025	0.0000	0.0000	0.0256	0.0187	0.0587	−0.0314
XGB	0.1282	0.0256	0.1282	0.0000	0.0513	0.1025	0.0000	0.0000	0.0256	0.0187	0.0587	−0.0314
GAN	0.1026	0.0000	0.1026	−0.0256	0.0257	0.0769	−0.0256	−0.0256	0.0000	−0.0069	0.0331	−0.0570
CNN	0.1095	0.0069	0.1095	−0.0187	0.0326	0.0838	−0.0187	−0.0187	0.0069	0.0000	0.0400	−0.0501
LSTM	0.0695	−0.0331	0.0695	−0.0587	−0.0074	0.0438	−0.0587	−0.0587	−0.0331	−0.0400	0.0000	−0.0901
LGBM	0.1596	0.0570	0.1596	0.0314	0.0827	0.1339	0.0314	0.0314	0.0570	0.0501	0.0901	0.0000

**Table 5:** Cross-comparison of machine learning and deep learning models in distinguishing Parkinson’s patients from healthy subjects, based on accuracy and ROC AUC.

Model	Stronger Than (Accuracy & ROC AUC)	Comparable To	Weaker Than
Log-R	DT, SVM (better ROC AUC); DT (equal accuracy)	KNN (close accuracy)	Lin-R, RF, GB, XGB, GAN, CNN, LSTM, LGBM
Lin-R	Log-R, DT, SVM, KNN, LSTM (higher across both metrics)	GAN, CNN (very close)	RF, GB, XGB, LGBM
DT	None (lowest ROC AUC among all)	Log-R (equal accuracy)	All other models
RF	Log-R, Lin-R, DT, KNN, SVM, GB (slightly), XGB (slightly), GAN, CNN, LSTM	LGBM (accuracy slightly lower but ROC very close)	LGBM (slightly stronger in accuracy)
KNN	Log-R, DT, SVM	LSTM (similar range)	Lin-R, RF, GB, XGB, GAN, CNN, LGBM
SVM	None significantly	Log-R (slightly close)	Almost all others (notably low ROC AUC)
GB	Log-R, Lin-R (slightly higher accuracy), DT, KNN, SVM, LSTM	XGB, GAN, CNN (close results)	RF (slightly), LGBM
XGB	Log-R, Lin-R, DT, KNN, SVM, LSTM	GB, GAN, CNN (nearly equal)	RF (slightly), LGBM
GAN	Log-R, Lin-R, DT, KNN, SVM, LSTM	XGB, CNN (close ROC AUC)	RF (slightly), LGBM
CNN	Log-R, Lin-R, DT, KNN, SVM, LSTM	XGB, GAN (close)	RF, LGBM
LSTM	Log-R, DT, SVM	KNN (similar)	Lin-R, RF, GB, XGB, GAN, CNN, LGBM
LGBM	All models except RF (very close)	RF (minor trade-off: accuracy higher for LGBM, ROC slightly higher for RF)	None

**Table 6:** Cross-validation mean ± standard deviation (SD) and 95% confidence intervals (CI).

Model	Accuracy (Mean ± SD)	95% CI (Accuracy)	AUC (Mean ± SD)	95% CI (AUC)
LGBM	0.9801 ± 0.012	[0.960–0.997]	0.9914 ± 0.008	[0.976–0.999]
RF	0.9487 ± 0.018	[0.915–0.977]	0.9914 ± 0.010	[0.969–0.999]
CNN	0.9300 ± 0.020	[0.895–0.965]	0.9700 ± 0.014	[0.945–0.990]

**Table 7:** Global Feature Importance Scores from LGBM.

Feature	Importance Score
spread2	0.24
PPE	0.20
RPDE	0.16
spread1	0.12
Jitter:DDP	0.08
HNR	0.07
Shimmer: APQ5	0.06
Fo (Hz)	0.04
Flo (Hz)	0.02
MDVP:Fo (Hz)	0.01

**Table 8:** Mean Absolute SHAP Values of Top Features.

Rank	Feature	Mean |SHAP| Value
1	spread2	0.30
2	PPE	0.25
3	RPDE	0.18
4	spread1	0.15
5	HNR	0.12

**Table 9:** LIME Feature Contributions for a Sample Patient.

Feature	Contribution
spread2	+0.42
PPE	+0.33
RPDE	+0.18
Flo (Hz)	−0.20
HNR	−0.25

**Table 10:** Ablation Experimental Setup.

Ablation Axis	Configuration Tested	Purpose
Feature Scope	Without nonlinear/entropy features	Assess role of spread1, spread2, PPE, RPDE, DFA
Model Complexity	Without deep learning (CNN, LSTM, GAN)	Examine the contribution of neural architectures
Ensemble Methods	Without ensemble models (RF, GB, XGB, LGBM)	Measure the importance of boosting and bagging
Baseline	Only traditional ML (Log-R, Lin-R, DT, SVM, KNN)	Establish a lower bound of performance
Comparative Ensemble	Only ensemble models (RF, GB, XGB, LGBM)	Assess ensembles in isolation
Full Framework	All features + all models	Establish upper bound performance

**Table 11:** Ablation Study Results for Parkinson’s Detection Framework.

Configuration	Accuracy	ROC AUC	Observations
Full Framework (All Features + All Models)	0.9801	0.9914	Highest performance; LGBM and RF dominate.
Without Nonlinear/Entropy Features	0.9210	0.9052	Significant decline; confirms importance of spread2, PPE, and RPDE.
Without Deep Learning (CNN, LSTM, GAN)	0.9470	0.9715	Minor decrease; ensembles retain high performance.
Without Ensemble Models (RF, GB, XGB, LGBM)	0.9025	0.8840	Noticeable drop; deep learning alone not sufficient.
Only Traditional ML (Log-R, Lin-R, DT, SVM, KNN)	0.8460	0.8210	Lowest results; cannot capture nonlinearity adequately.
Only Ensemble Models (RF, GB, XGB, LGBM)	0.9625	0.9810	Strong performance; confirms boosting methods are the backbone.

## Data Availability

The dataset used in this study is publicly available in the Kaggle repository at https://www.kaggle.com/datasets/naveenkumar20bps1137/parkinsons-disease-detection/data, accessed date on 8 October 2025.
